# Characterization of Optimal Optogenetic Stimulation Paradigms to Evoke Calcium Events in Cortical Astrocytes

**DOI:** 10.1523/ENEURO.0220-25.2025

**Published:** 2025-09-12

**Authors:** Lakshmini Balachandar, Carolina Moncion, Alejandro Suarez, Jorge Riera Diaz

**Affiliations:** NMD Laboratory at the Department of Biomedical Engineering, Florida International University, Miami, Florida 33174

**Keywords:** astrocyte, calcium imaging, cortex, optogenetics, transgenic mice

## Abstract

Understanding the roles of astrocytic calcium signaling in multiple brain regulatory mechanisms including metabolism, blood flow, neuromodulation, and neuroinflammation has remained one of the enduring challenges in glial biology. To delineate astrocytic contribution from concurrent neuronal activity, it is vital to establish robust control and manipulate astrocytes using a technique like optogenetics due to its high cellular specificity and temporal resolution. The lack of an experimental paradigm to induce controlled calcium signaling in astrocytes has hindered progress in the field. To address this, in this study, we systematically characterize and identify light stimulation paradigms for inducing regulated, on-demand increases in astrocytic calcium in acute brain slice cortical astrocytes from MlC1-ChR2(C128S)-EYFP mice (of either sex). We identified paradigms 20, 40 and 60% (of *T* = 100 s) to elicit robust calcium responses upon periodic stimulations, while the 95% paradigm exhibited a response only during the first stimulation. We also quantified several parameters, including peak height, full-width at half-maximum (FWHM), and latencies, and observe that the 20% paradigm/duty cycle has the highest peak Δ*F*/*F*_0_ among the paradigms across all stimulations and the lowest FWHM during the first stimulation. To illustrate the impact of our study, we observed robust changes in cerebral blood flow, because of 20% optogenetic stimulation, in vivo, using laser Doppler flowmetry. Overall, the 20% paradigm is a favorable choice for eliciting robust astrocytic calcium responses in astrocytes while performing periodic stimulations.

## Significance Statement

Reactive astrogliosis observed in several neurological disorders is associated with neuroinflammation and enhanced astrocytic calcium levels. The multicellular nature of the neuroinflammatory milieu poses challenges in deciphering the exact role of astrocytic calcium signaling. To address the lack of a characterized method in using optogenetics for eliciting astrocytic calcium increases, and based on a recent in silico study by Moshkforoush et al. (2021), we identified light stimulation paradigms resulting in consistent astrocytic calcium increases in acute brain slice preparations. We also demonstrate that the favorable paradigm for inducing astrocytic calcium increases leads to robust increases in cerebral blood flow in vivo. This study thereby investigates and identifies light stimulation paradigms for achieving tunable astrocytic calcium changes via periodic stimulations.

## Introduction

Astrocytes are an abundantly present glial cell type tiling the central nervous system (CNS) and have crucial functions. These include maintenance of the blood–brain barrier (Abbott, 2002; Heithoff et al., 2021), role in neuronal migration (Carmen et al., 2007), providing structural, metabolic support, and plasticity to neurons (Theodosis et al., 2008; Rose et al., 2013; Bernardinelli et al., 2014), and neurotransmitter homeostasis (Murphy-Royal et al., 2017), compose the neuro-glio-vascular unit, regulate cerebral blood flow (CBF; Takano et al., 2006; Attwell et al., 2010; Lind et al., 2013), and form the quad-partite synapse with microglia and neurons (Schafer et al., 2013). Astrocytes modulate neuroinflammation AND undergo morpho-functional and molecular changes in several neurological disorders, thereby becoming reactive and existing in multiple states (Seifert et al., 2006; Sofroniew, 2009; Vezzani et al., 2011). Dysregulated astrocytic calcium signaling has been observed as a hallmark in several neurological diseases including Alzheimer's disease (Kuchibhotla et al., 2009; Shigetomi et al., 2019) and epilepsy (Ding et al., 2007). Probing the exact role of astrocytic calcium signaling and its involvement in the generation of neuroinflammation can offer answers to delineate astrocytic contribution in health and disease.

Optogenetics facilitates genetically targeting and incorporating light-sensitive ion channels like channelrhodopsin 2 (ChR2) into specific cell types with high temporal resolution, cellular specificity, and spatial precision (Adamantidis et al., 2014; Deisseroth, 2015). In a recent in silico study by Moshkforoush et al. (2021), it was computationally demonstrated that increasing optogenetic light stimulation at a given time led to increases in astrocytic calcium responses, until a particular threshold, beyond which the levels of astrocytic calcium began to decline. For short periods of optogenetic stimulation in astrocytes (in the order of minutes), it was demonstrated computationally that at a given time period, an increase in duration of stimulation/duty cycle initially led to an increase in calcium spiking, but subsequently led to decreases in astrocytic spiking activity, potentially due to depletion of ER stores, buffer proteins, and SERCA and PMCA pumps. This was shown to be in congruence with a continual increase in astrocytic calcium baseline levels due to calcium influx due to ChR2, before saturation at high durations of stimulation. Additionally, while Shen et al. (2017) briefly demonstrated two repeated optogenetic stimulations with a rest period in astrocytes, Cho et al. (2022) show increases in intracellular astrocytic calcium signals for a few seconds of stimulation. However, a holistic approach to characterize and quantify the effect of repeated optogenetic stimulation in astrocytes to evoke astrocytic Ca^2+^ increases is yet to be performed, unlike in neurons. In a recent study from Suarez et al., optogenetic stimulation of ChR2-expressing astrocytes was shown to lead to changes in CBF (both rapid and slow responses; Suarez et al., 2025).

In this manuscript, we experimentally test the findings of the in silico model in Moshkforoush et al. (2021) for short-term optogenetically induced astrocytic calcium signaling and evaluate several experimental paradigms. We systematically evaluate the effect of repeated light stimulation in mice cortical astrocytes in acute brain slice preparations from a knock-in murine model for optogenetic control of astrocytes. Mlc1-tTA::tetO-ChR2(C128S)-EYFP mice developed by Tanaka et al. (2012) offers an expression of channelrhodopsin 2 [ChR2(C128S)], the bistable opsin in astrocytes, viz., the MlC1 promoter. We identify paradigms that lead to robust astrocytic responses with every light incidence and characterize several astrocytic Ca^2+^ signaling parameters to compare the effect of light stimulation paradigms.

**Figure 1.**
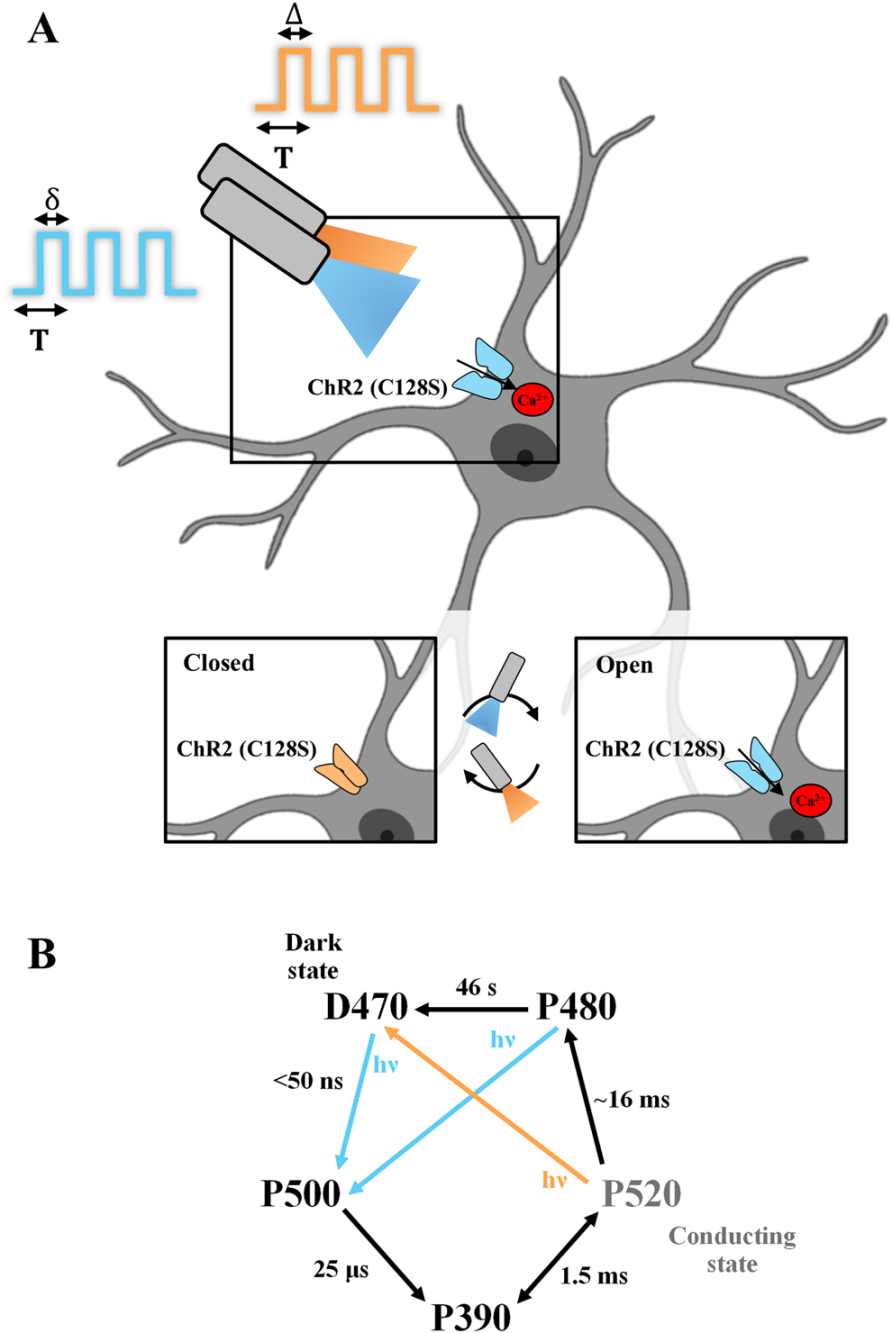
Schematic of experimental paradigms for simultaneous optogenetic stimulation and astrocytic calcium imaging. ***A***, Expression of Mlc1-ChR2(C128S)-EYFP on the astrocytic membrane allowing the influx of Ca^2+^ upon blue light stimulation, inset shows closed and open conﬁgurations of ChR2 on the astrocytic membrane, opened by blue light to allow an inﬂux of Ca^2+^ and closed by amber light. *T* denotes the pulse period of light stimulation and δ (blue) and Δ (amber) denote pulse widths within *T* for which light is on. Created using BioRender. ***B***, Photocycle of ChR2(C128S), adapted from Berndt et al. (2009); Ritter et al. (2008) with corresponding time conversions.

In this study, *T* denotes the pulse period of light stimulation and δ_blue_ (for blue light) and Δ (for Amber light) denote pulse widths within *T*/duty cycle for which light is on (Fig. 1). We define a paradigm to be the value of δ within the duration of *T* = 100 s for which the blue light is on [20% paradigm/duty cycle refers to δ of 20 s/20% of *T* (100 s)]. We identified paradigms (δ_blue_) of 20, 40 and 60% (of *T* = 100 s) to elicit robust Ca^2+^ responses upon periodic stimulations, 80% leading to a reduction in astrocytic calcium response levels, while the 95% paradigm exhibited a response only during the first stimulation. We quantified several parameters, including peak height, full-width at half-maximum (FWHM), and latencies, and observe that the 20% paradigm has the highest peak Δ*F*/*F*_0_ among the paradigms across all stimulations and the lowest FWHM during the first stimulation. To demonstrate the impact of our study, we observed that the 20% paradigm led to robust changes in CBF in MlC1^+^ChR2^+^ mice, quantified using laser Doppler flowmetry (LDF), in vivo. Overall, the 20% paradigm is a favorable choice for eliciting robust astrocytic Ca^2+^ responses in astrocytes while performing multiple stimulations, followed by 40%, although with lesser Δ*F*/*F*_0_. Collectively, the framework presented in our study provides valuable information in the identification and selection of light stimulation paradigms resulting in consistent astrocytic calcium increases across stimulations and could help in delineating astrocytic contributions from concurrent neuronal activity.

## Materials and Methods

All experimental procedures and animal care in this study were approved by the Institutional Animal Care and Use Committee (IACUC; Approval No. 19-045, 22-049), IBC exception protocol (18-006), and NIH ARRIVE guidelines. tTA-MlC1-tetO-ChR2(C128S)-EYFP (of either sex) mice were employed in this study, which were bred in house after reviving embryos from RIKEN, Japan (RBRC05450 and RBRC05454). These mice were housed in standard cages at a 12 h light/dark cycle with *ad libitum* access to food and water. They express ChR2 and EYFP exclusively in astrocytes (using the MlC1 promoter) and were used in both acute brain slice preparations and in vivo experiments.

### Acute brain slice experiments

#### Slice preparation

The detailed protocols and methodology for acute brain slice preparation, optogenetic stimulation, image acquisition, and analysis are as per a previous study by Balachandar et al. (2020). Briefly, acute brain slices were prepared from Mlc1-ChR2(C128S)-EYFP mice (either gender, 2–5 months of age) in a sucrose-based, ice-cold cutting solution with carbogen (95% O_2_, 5%CO_2_), using a vibratome (Vibratome 1000 Plus). Slices were placed in a recovery solution at 34°C with active bubbling of carbogen for 30 min, followed by an additional 30 min at room temperature (RT). The brain slices were then stained with 5.7 μM Rhod-2 AM (in 10% Pluronic and 5% Kolliphor EL/DMSO) at 34°C in a water bath for 45 min. Slices were subsequently washed and stored at RT in aCSF while imaging.

#### Optogenetic stimulation and calcium imaging

Prior to calcium imaging, an EYFP and Rhod-2 AM coregistered image was acquired for every field of view, to enable selection of Rhod-2 AM loaded astrocytes during analysis. Rhod-2 AM loaded astrocytes were imaged at least 20 μm beneath the surface of the slices (to avoid superficial reactive cells due to tissue cutting/processing). The overlap between ChR2(C128S)-EYFP and Rhod-2 AM channels was used to determine astrocytes loaded with Rhod-2 AM calcium indicator. Time-lapse imaging using confocal microscopy of Mlc1-ChR2 (C128S)-EYFP mice brain slices loaded with Rhod-2 AM is performed to evaluate astrocytic Ca^2+^ responses upon light stimulation (Balachandar et al., 2020). Simultaneous whole-field optogenetic stimulation (500 μW power, corresponding to 7 μW/mm^2^ power density for the 470 nm fiber-coupled LED/blue, and 100 μW for the 595 nm fiber-coupled LED/amber, measured using Thorlabs PM100A with S121C sensor) and calcium imaging of astrocytes were synced and performed using a 10X water objective (Olympus UMPlanFLN), using an Olympus FV 1200 confocal microscope (with filter cubes U-N49002 and U-N49004). The illumination area encompassed the entire slice (was not tightly focused), as the goal was to broadly illuminate astrocytes within the field of view during calcium imaging. The distance at which the LEDs were placed was confirmed before each experiment by using the power meter sensor placed where the brain slices would be placed, to ensure reproducibility.

Figure 1*A* shows closed and open configurations of Mlc1-ChR2(C128S)-EYFP on the astrocytic membrane, opened by blue light to allow an influx of Ca^2+^ and closed by amber light. Each stimulation paradigm was programmed using MATLAB and the corresponding Data Acquisition Toolbox. The confocal acquisition trigger signal received with the NI-DAQ TTL cable every time an image was acquired was used to synchronize imaging and the programmed stimulation paradigm. With these synchronized signals, it was possible to appropriately trigger the *T*-cube LED drivers via a BNC cable to stimulate. Figure 1*B* also illustrates the channel kinetics of ChR2(C128S) and corresponding time conversions. Differing blue light stimulation paradigms (δ_blue_) were employed in this study—the blue light stimulation paradigms were chosen to be a fraction of the paradigm period (*T* = 100 s)/duty cycle, 20, 40, 60, 80, and 95%, and five stimulations of each paradigm were incident on the brain slices. Each of these blue light stimulations was followed by a 5 s amber pulse (Δ = 5% of *T*).

#### Data analysis

**Figure 2.**
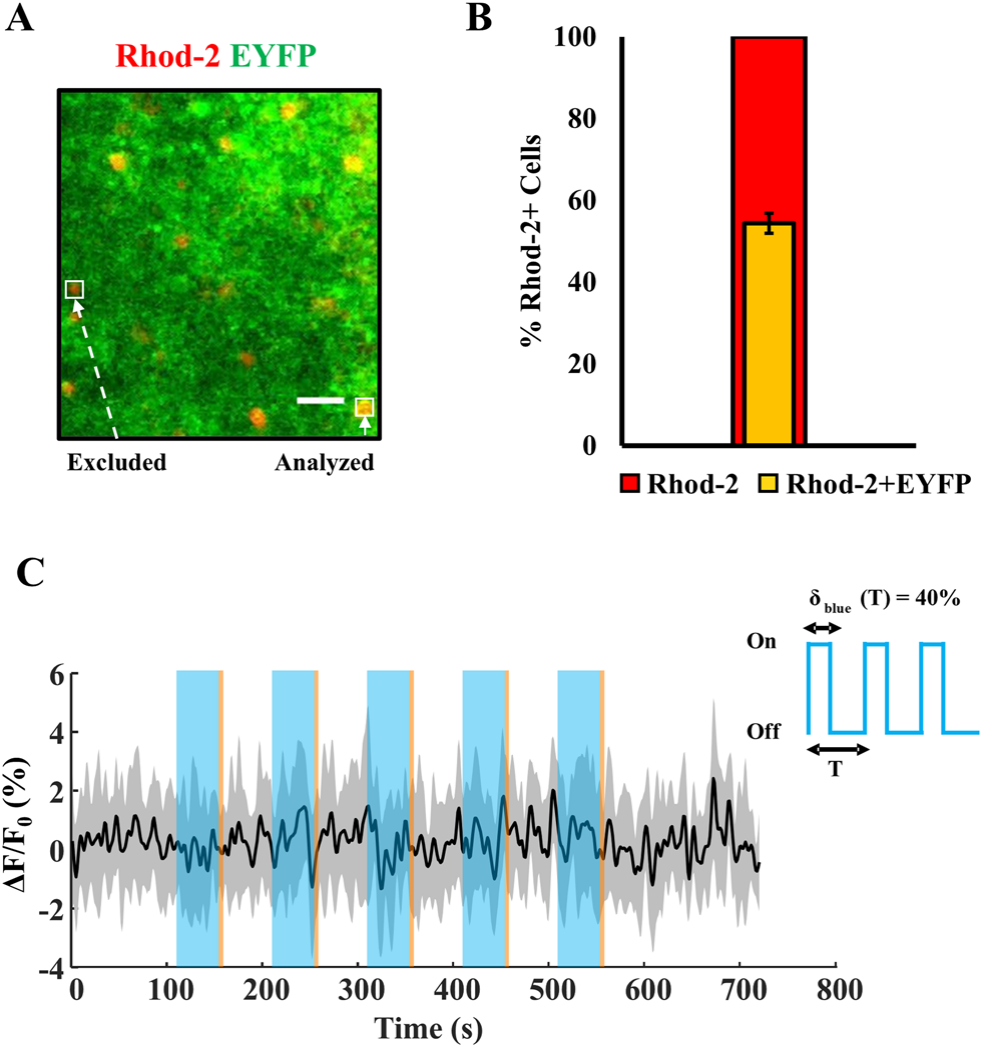
Quantification of astrocytic staining and calcium responses from a WT mouse. ***A***, Left, Representative image of a cortical field of view from mice expressing Mlc1-ChR2(C128S)-EYFP (green), showing overlap with Rhod-2 AM staining (red). ***B***, A fraction of Rhod-2 AM positive cells expressing Mlc1- ChR2(C128S)-EYFP in the neocortex, indicating astrocytes (*n* = 8 mice, 10 sections, mean ± SD). Scale bar, 30 μm. (Adapted from Balachandar et al. (2020), © 2020 Wiley Periodicals LLC). ***C***, Average Δ*F*/*F*_0_ trace of Rhod-2 AM positive cells in a wild-type C57BL6/J control mouse (JAX labs), *n* = 15 cells, 1 mouse. Light stimuli: blue, 40 s/500 μW [i.e., δ_blue_(*T*) = 40%; *T* = 100 s] and amber, 5 s/100 μW.

After the time-series data was collected, motion correction [using NoRMCorre (Pnevmatikakis and Giovannucci, 2017)] was implemented. Videos were further analyzed using FIJI ROI analyzer (Schindelin et al., 2012) where regions of interest (ROIs; astrocytes) were selected, based on the inclusion criteria of EYFP-Rhod-2 AM overlap (as illustrated in Fig. 2*A*). Astrocytes which were loaded with Rhod-2 AM and having EYFP labeling were chosen for downstream analysis. A *Z*-project image was used to analyze the most active/responsive cells from each slice. Time-series data for each astrocyte (ROI) was generated on FIJI and imported to MATLAB for detrending, smoothing, visualization of traces as Δ*F*/*F*_0_ [baseline fluorescence (*F*_0_) is taken to be the median fluorescence intensity of the prestimulus imaging window], and extraction of signal properties (peak response, FWHM etc.). Sample size is as mentioned in the corresponding figure captions (number of mice, number of slices, or number of cells). Statistical analysis *t* tests and two-way ANOVA with interactions were performed on GraphPad Prism v9.2.

### In vivo experiments

#### Surgical procedure

Transgenic mice (10–12 weeks, *n* = 4) were anesthetized using isoflurane (1.5–2.5% 100% O_2_ 1 L/min, 14.7 psi) and restrained in a stereotaxic apparatus. Anesthesia was monitored by keeping the body temperature ∼37 ± 0.5°C using a heating pad, and respiration rate was maintained ∼60 ± 10 BPM. After confirming the lack of righting reflex, the scalp was shaved, skull was exposed, and a craniotomy of 2–3 mm diameter was performed to expose the cortex. Following the surgical procedures, a bolus injection of dexmedetomidine (0.05 ml) was administered intraperitoneally, along with reduction of the level of isoflurane to 0.5% in order to reduce vascular effects of the anesthetic (You et al., 2021).

#### Optogenetic stimulation and blood flow changes

Similar to our acute brain slice experiments, two fiber-optic LEDs, blue (470 nm wavelength, 0.15 mW/mm^2^) and amber (595 nm wavelength, 0.01 mW/mm^2^), were employed to open and close ChR2, respectively. The 20% paradigm of blue light stimulation was chosen where 470 nm (blue) light was on for 20 s, followed by 5 s of 595 nm (amber) light, followed by a dark phase of 75 s (*T* = 100 s). For each mouse, five paradigm periods were recorded. LDF (PeriFlux 4001 Master) was used to measure regional changes in CBF induced by optogenetic stimulation. An LDF probe (needle probe 411, Perimed) was placed using a micromanipulator (MPC-325, Sutter Instrument) and guided by a digital microscope (VCR-800, HIROX) until a small deflection of the dura was observed on top of the exposed cerebral cortex. At this location, probe advancement ceased, and the probe was retracted by 20–50 μm. The probe was moved to find an area with maximum blood flow fluctuations and avoiding interferences from larger vessels. Animals were killed after recording completion.

#### Data acquisition and processing

Localized cerebral blood perfusion data and the LED (Blue and Amber) stimulation triggers (voltage signal, 5 V amplitude) were simultaneously recorded by using PowerLab (ADInstruments) and exported to MATLAB. LDF and stimulation trigger data were downsampled to 10 data points per second (10 Hz), and frequencies over 0.1 Hz were removed from LDF data using a low-pass filter (Butterworth, fifth order). The evoked CBF response was obtained by averaging the response from all mice in response to optogenetic stimulation. Since LDF measurements do not reflect the absolute perfusion value and vary with each mouse, data segments were normalized to the baseline perfusion value. This value was determined by averaging the prestimulation data of LDF (117 s). The data is represented as the mean with standard deviation.

## Results

### Response of astrocytic calcium to varying light stimulation

**Figure 3.**
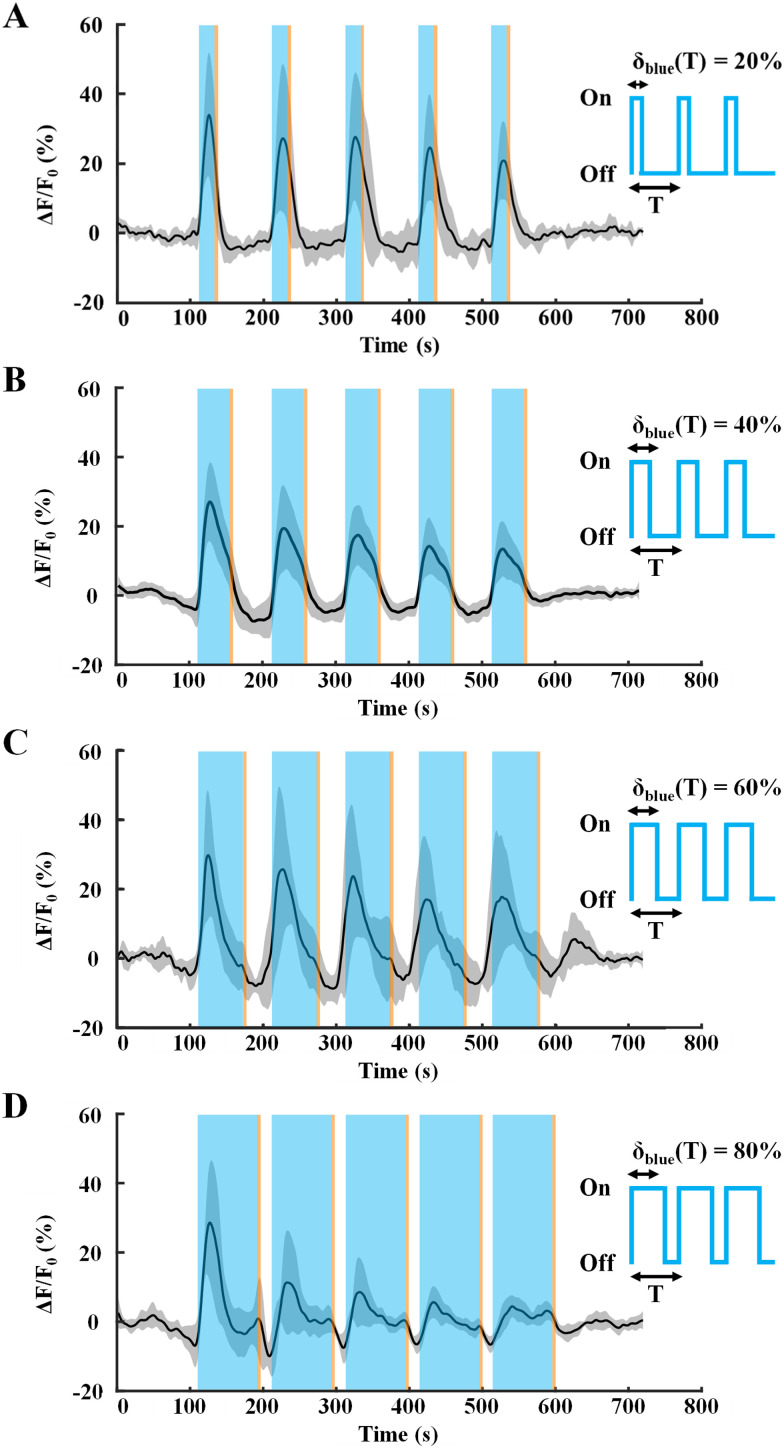
Light-evoked Rhod-2 AM calcium responses from adult murine cortical astrocytes subjected to various light stimulation paradigms. ***A***, Average Δ*F*/*F*_0_ trace (central dark trace) of calcium responses from cortical astrocytes expressing Mlc1-ChR2(C128S)-EYFP (*n* = 14 cells, 3 slices, 3 mice) upon repeated light stimulation over the recording period, and gray shaded region depicts the standard deviation. Blue and amber shaded bars represent wavelengths of LED illumination of the brain slice during light stimulation. Light stimuli: blue, 20 s/500 μW [i.e., δ_blue_(*T*) = 20% where *T* = 100 s] and amber, 5 s/100 μW pulses were produced using LED light sources to open and close ChR2(C128S) channels, respectively (denoted as shaded blue and amber colored regions). ***B***, Average Δ*F*/*F*_0_ trace of astrocytes, *n* = 37 cells, 3 slices, 3 mice. Light stimuli: blue, 40 s/500 μW [i.e., δ_blue_(*T*) = 40%; *T* = 100 s] and amber, 5 s/100 μW. ***C***, Average Δ*F*/*F*_0_ trace of astrocytes, *n* = 18 cells, 5 slices, 5 mice. Light stimuli: blue, 60 s/500 μW [i.e., δ_blue_(*T*) = 60%; *T* = 100 s] and amber, 5 s/100 μW. ***D***, Average Δ*F*/*F*_0_ trace of astrocytes, *n* = 15 cells, 3 slices, 3 mice. Light stimuli: blue, 80 s/500 μW [i.e., δ_blue_(*T*) = 80%; *T* = 100 s] and amber, 5 s/100 μW. Refer to Extended Data Figure 3-1 for near continuous light stimulation [δ_blue_(*T*) = 95%] trace.

Figure 3 shows the light-evoked response of MlC1^+^/Rhod-2 AM^+^ astrocytes subjected to various blue light stimulation paradigms/duty cycles. The blue light stimulation paradigms (δ_blue_) were chosen to be a fraction of the paradigm period (*T* = 100 s), 20, 40, 60, and 80%, and five stimulations of each paradigm were incident on the brain slices. Each of these blue light stimulations was followed by a 5 s amber pulse (5% of *T*). While repeated light stimulation of 20, 40, and 60% (Fig. 3*A–C*) elicited robust calcium responses in these neocortical astrocytes, the 80% paradigm (Fig. 3*D*) led to a decline in peak responses over increasing stimulation number and the 95% paradigm led to only one calcium elevation (Extended Data Fig. 3-1) followed by minimal to no response during the subsequent stimulations.

10.1523/ENEURO.0220-25.2025.f3-1 Figure 3-1 Astrocytic calcium responses to near continuous light stimulation. Download Figure 3-1, TIF file.

It is important to note three aspects of these calcium responses—firstly, all calcium responses tend to initially increase with light stimulation due to cytosolic calcium influx via ChR2 and with time, start decreasing. This can be attributed to the bell-shaped response of the IP_3_R to cytosolic calcium, where an influx of calcium into the astrocytic cytosol leads to translocation of calcium from the ER to cytosol but subsequently, at higher cytosolic concentrations leads to inhibition of the IP_3_R, resulting in no subsequent calcium spikes (Watras and Ehrlich, 1991; Mak and Foskett, 2015). Secondly, calcium clearing mechanisms via buffer proteins, sarco(endo)plasmic reticulum Ca^2+^-ATPase (SERCA) and plasma membrane Ca^2+^ ATPase (PMCA) pumps and the mitochondrial Ca^2+^ uniporter (MCU) also reduce cytosolic calcium. Lastly, it is also important to note that when blue light is on, the short-lived conducting P520 state of ChR2(C128S) exists in equilibrium with the P390 state and could also transition to the P480 and P500 states, which are nonconducting (Fig. 1*B*; Ritter et al., 2008; Berndt et al., 2009).

This could also lead to a reduction of influx of calcium into the cytosol via ChR2, despite blue light being on, and in combination with the abovementioned mechanisms, may result in lowering the net cytosolic calcium. It is also crucial to note that the effect of blue light causing robust astrocytic calcium responses is observed only in MlC1^+^-ChR2^+^ mice, whereas in WT controls (Fig. 2*C*), there are no changes to the astrocytic calcium baseline, thereby corroborating the dynamic nature of the optogenetic transgenic mouse model employed in this study.

### Quantification of peak characteristics across paradigms and stimulations

**Figure 4.**
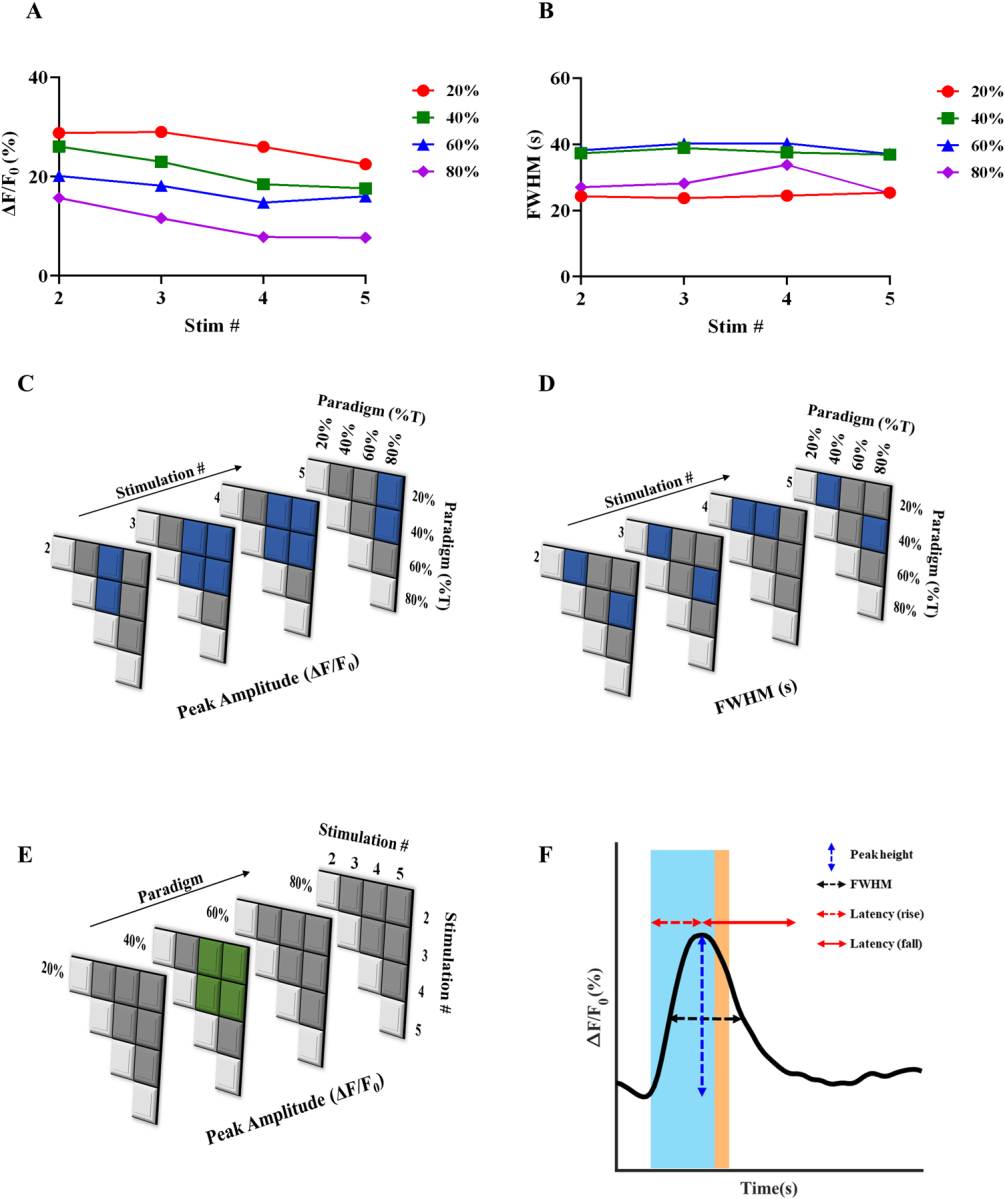
Quantification of astrocytic calcium responses (peak height and FWHM) from acute brain slices subjected to blue stimulation. ***A***, Scatterplot quantifying means of astrocyte peak responses–peak height Δ*F*/*F*_0_, across stimulation paradigms and stimulation number. ***B***, Heat map showing statistically significant differences in peak height (shaded boxes) across stimulation number and (***C***) across paradigms. ***D***, Scatterplot quantifying means of FWHM of astrocytic calcium responses, across stimulation paradigms and stimulation number. ***E***, Heat map showing statistically significant differences in FWHM (shaded boxes) across stimulation number. Significance calculated at 95% confidence. ***F***, Illustration of parameters quantified in this study pertaining to astrocytic calcium response–peak height, FWHM, and latencies of rise and fall.

Figure 4*F* illustrates the parameter quantification performed for the subsequent sections. They are (1) peak height (Δ*F*/*F*_0_) within a stimulation paradigm, (2) FWHM, (3) latency rise (time taken from the onset of stimulation until peak height is achieved), and (4) latency fall (time taken from peak height until calcium basal level, matching prestimulation level is achieved).

A comparison across light stimulation paradigms helps us understand the trends associated with progressing from a lower (20%) to a higher stimulation paradigm (80%). The peak height (Δ*F*/*F*_0_) has a declining trend from the 20–80% paradigm across all the stimulations (Fig. 4*A*). Figure 4, *B* and *C*, shows that with increasing stimulation number (3, 4), there are more significant differences between the lower set of stimulation paradigms (20–60%) and the higher stimulation paradigms (80 and 95%), as compared with stimulations 2 and 5. As mentioned in the previous section, we speculate that due to prolonged light stimulation, ChR2(C128S) is driven toward its nonconducting states, and coupled with the inhibition of the IP_3_R, the buffering, SERCA, PMCA pumps, and MCU, there is a net reduction in cytosolic calcium beyond a point of time. The lack of a prolonged dark phase prevents ChR2(C128S) from returning to its D470 state, which is photosensitive and could progress to the conducting state, resulting in the lack of robust responses in stimulations 2–5. While comparing peak Δ*F*/*F*_0_ across various stimulation for a given paradigm, 40% has the highest significant differences across stimulations (Fig. 4*C*).

Figure 4*D* shows that there is an initial increase in FWHM with increasing paradigm (20–40/60%), indicative of increased sustenance of astrocytic calcium levels before returning to baseline. However, when light stimulation is further increased to 80%, there is a reduction in FWHM, potentially due to inactivation of the IP_3_R, resulting in saturation of calcium levels and subsequent reduction and return to baseline upon constant stimulation. Figure 4*E* shows that there is a consistent significant difference between 20 and 40% paradigms across all stimulation numbers and significant differences between 40 and 80% in almost all stimulation numbers.

### Quantification of latencies across paradigms

**Figure 5.**
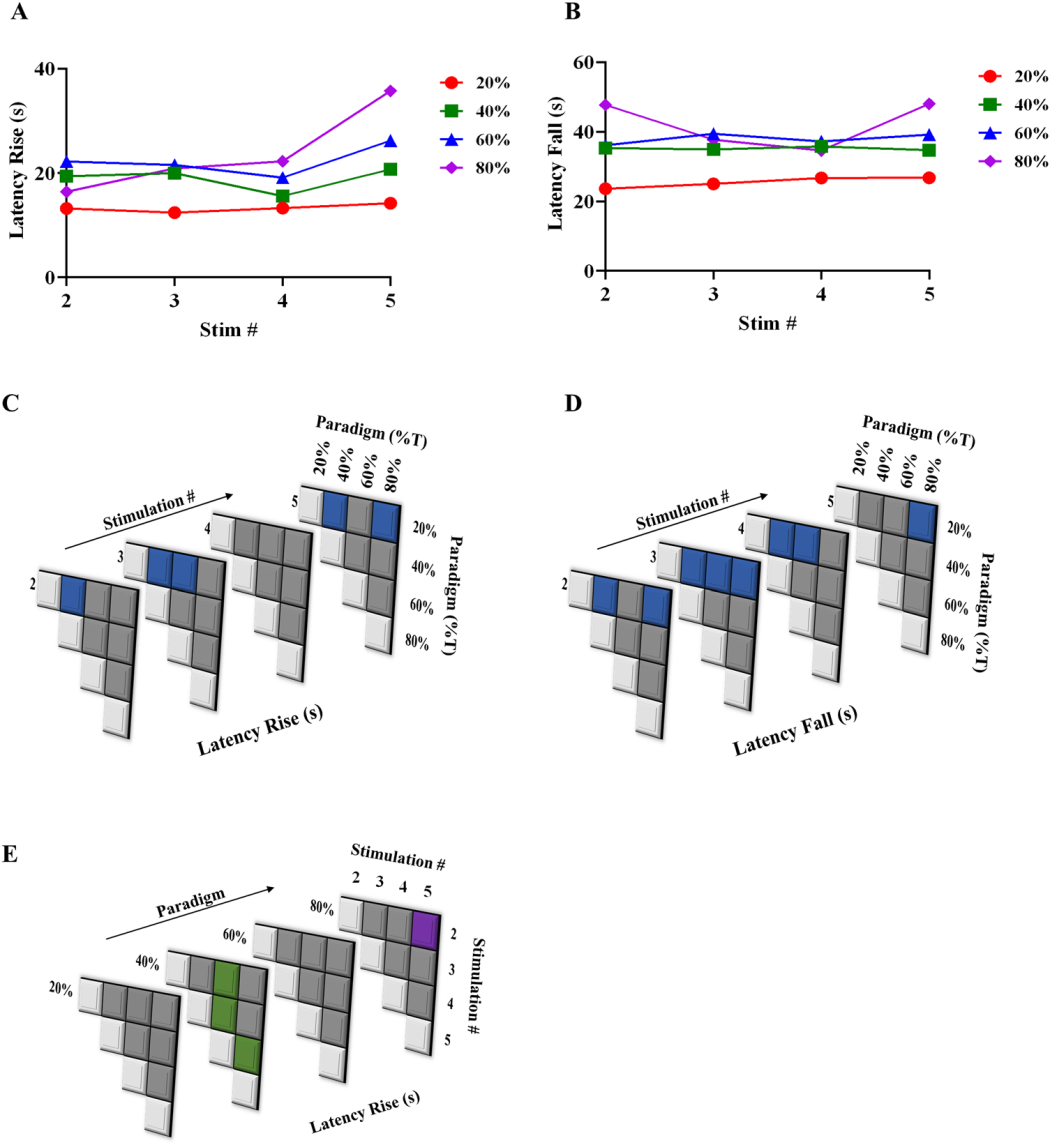
Quantification of astrocytic calcium responses (latencies) from acute brain slices subjected to blue stimulation. ***A***, Scatterplot quantifying means of astrocyte peak responses–latency of rise, across stimulation paradigms and stimulation number. ***B***, Heat map showing statistically significant differences in latency rise (shaded boxes) across stimulation number and (***C***) across paradigms. ***D***, Scatterplot quantifying means of latency of fall of astrocytic calcium responses, across stimulation paradigms and stimulation number. ***E***, Heat map showing statistically significant differences in latency fall across stimulation number. Significance calculated at 95% confidence.

Quantification of the latency (rise and fall) displays interesting trends compared with paradigms and repeated stimulations (Fig. 5*A*,*D*). With the quantification of rise parameters, the latency during the earlier stimulations—stimulation 2 showing an increasing trend from 20 to 60%, and a lower latency for 80%, while at the later stimulations (4 and 5) show a consistent increase from paradigms 20–80% (Fig. 5*A*). This is in accordance with our results in the previous section—wherein longer periods of stimulation (increasing paradigm) could lead to accumulation of calcium, and not enough time for calcium scavenging mechanisms in the astrocyte to reduce intracellular calcium levels. Figure 5*B* shows that with increasing stimulations, the significant changes in latency rise periods initially increased until stimulation 3, skipped 4 and resumed in 5. Within a given paradigm, 40% saw maximum significant differences across stimulations, followed by 80% (Fig. 5*C*). Figure 5, *D* and *E*, show that the latency of fall was significantly increased at 80%, as compared with 20% at the earlier stimulation, becomes comparable with 40 and 60% at subsequent stimulations, and again becomes significant at stimulation 5.

### Optogenetic stimulation of astrocytes results in CBF changes in the cortex of transgenic mice

**Figure 6.**
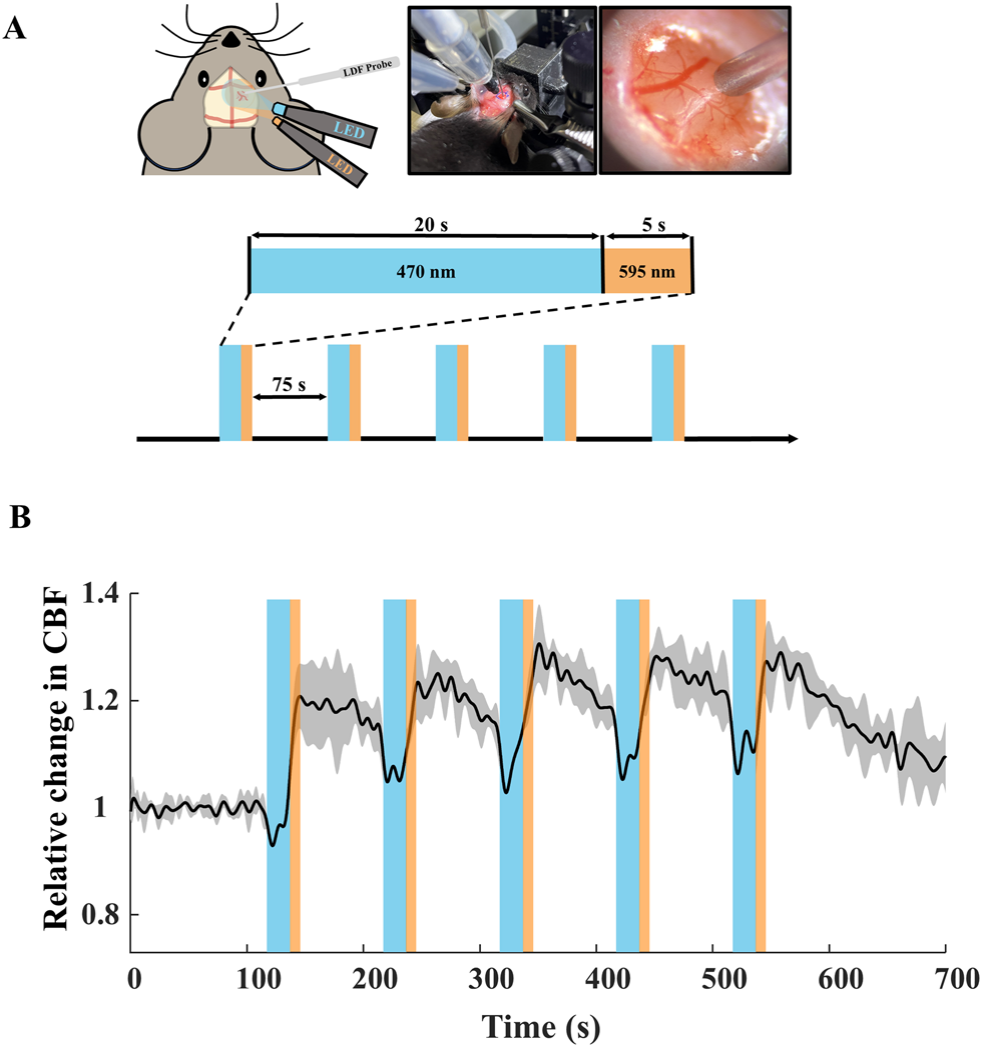
Quantification of CBF changes using LDF, as a result of optogenetic stimulation in MlC1^+^-ChR2^+^ mice. ***A***, Schematic of simultaneous optogenetic stimulation and LDF recordings, along with an example of LDF probe placement over the mouse cortex. The 20% stimulation paradigm [δ_blue_(*T*) = 20% where *T* = 100 s] was used for this in vivo evaluation of CBF. ***B***, Relative CBF response (black trace) ± SD (gray shaded region) as a result of 20% blue light stimulation (five consecutive optogenetic stimulation periods (*T*; blue shaded bars) pulses of 20 s of blue light followed by 5 s of amber light pulses). *N* = 4 mice.

Figure 6*A* shows the setup and schematic of the Mlc1^+^ChR2^+^ mouse on which optogenetic stimulation using 470 and 595 nm LEDs and recording using an LDF probe are being performed. The zoomed section shows an example field of view for LDF recordings. Below is an illustration of the optogenetic stimulation paradigm. Figure 6*B* shows an increase in CBF in response to optogenetic stimulation of astrocytes. Upon incidence of blue light (470 nm) during the first stimulation, a CBF response rises and does not quite return to baseline. This could be due to the delay in kinetics of the hemodynamic response as compared with calcium imaging. Upon cessation of blue light, CBF reduces during the 75 s of the dark/no light phase but subsequently increases again upon the next blue stimulation. After five cycles of blue-amber light, the CBF begins to return to baseline. This response is synchronized with our acute brain slice preparations (Fig. 3), where we observe robust astrocytic calcium increases upon stimulation with repeated blue light pulses of 20 s each (*T* = 100 s).

## Discussion

Several research groups have demonstrated the ability to elevate calcium activity in astrocytes via electrical (Fleischer et al., 2015; Monai et al., 2016; Monai and Hirase, 2016), mechanical (Charles et al., 1991; Guthrie et al., 1999; Stout et al., 2002), and pharmacological (Parpura et al., 1994; Jeremic et al., 2001; Durkee et al., 2019) approaches. Electrical stimulation lacks cell specificity due to potential concurrent activation of neurons and suffers low spatial resolution. Mechanical stimulation, performed to mimic responses to brain injury and spreading depression (Ellis et al., 1995; Nedergaard et al., 1995), does not allow the study of astrocytic functions under normal physiological conditions. The use of pharmacological techniques for targeting these cells in the brain has been limited to basic research due to high invasiveness and low temporal resolution. On the other hand, optogenetics is a platform facilitating genetic targeting of cells in a high spatial and temporal manner, which can be used to delineate astrocytic involvement in multicellular phenomena in the CNS, from concurrent neuronal activity (Fenno et al., 2011; Krook-Magnuson et al., 2014).

A wide range of optogenetic tools have been constructed despite the recent inception of the field of optogenetics, among which ChR2 has been one of the most used. It is an algal-derived light-sensitive cationic channel, which undergoes a conformational change from all-trans to cis configuration upon exposure to blue light (Nagel et al., 2003). ChR2 constructs have also been modified to create bistable opsins, where photocurrents via the channel can be initiated and terminated precisely with different wavelengths of light. An example is the ChR2(C128S) variant which is ∼300 times more sensitive to light than the wild-type variant, with long-term activation and precise switching between the open and closed states of the channel (Berndt et al., 2009). Recently, Cho et al. demonstrated that astrocytic calcium modulation using optogenetics led to post-stroke recovery (Cho et al., 2025), which was done using OptoSTIM1. This was by employing targeted endogenously expressed calcium release-activated calcium channels, unlike the conventional opsin-based optogenetic tools. Optogenetics has also helped understand astrocytic roles in contextual fear memory (Li et al., 2020), in rescuing stress-induced anxiety (Xiao et al., 2020), modulation of spike-wave discharges in absence epilepsy (Ozgur et al., 2022), and in restoring slow brain rhythm function in AD pathology (Lee et al., 2023), thereby shedding light on possible therapeutic approaches. Optogenetic intervention of astrocytes coupled with theoretical evaluations can help further the understanding of the intricacies in astrocytic responses to light and help establish robust control and applied in translational animal studies (Moshkforoush et al., 2021; Balachandar et al., 2022). Hence, control of astrocytes via modulation of Ca^2+^ oscillations using techniques like optogenetics can prove to be crucial in therapeutic intervention of a variety of neurological disorders.

In this study, we identify light stimulation paradigms leading to robust neocortical astrocytic calcium increases in short-term stimulation. We identified paradigms 20, 40 and 60% (of *T* = 100 s) to elicit robust calcium responses upon periodic stimulations, while the 95% paradigm exhibited a response only during the first stimulation (Extended Data Fig. 3-1). We quantified several parameters, including peak height, FWHM, and latencies, and observe that the 20% paradigm has the highest peak Δ*F*/*F*_0_ among the paradigms across all stimulations and the lowest FWHM during the first stimulation. Overall, the 20% paradigm is a favorable choice for eliciting robust astrocytic calcium responses in astrocytes while performing multiple stimulations. We also corroborate this, using LDF recordings in vivo, where we show robust increases in CBF as a response to 20% light stimulation.

While stimulating acute brain slice preparations with the 95% paradigm, it is interesting to note that after the first blue-amber stimulation, there are no major calcium elevations during the subsequent stimulations (Extended Data Fig. 3-1). There appears to be a stealth inactivation of the astrocytes as stimulation is continued, the exact mechanism of which is unknown. In addition to calcium clearing cellular mechanisms in the astrocyte, we speculate about the possibility that due to lack of time for achieving the light-sensitive D470 state from P480 (∼46 s) due to no dark phase throughout the stimulation (due to 95 s of blue followed by 5 s of amber), ChR2(C128S) could exist in the nonconducting states, thereby maintaining its calcium levels comparable with prestimulation (Fig. 1*B*, Extended Data Fig. 3-1). Whether this inactivation can be overcome by introducing a rest period of a few minutes between each 95% stimulation is yet to be explored, which would require an increase in recording times. Interestingly, in neurons, upon stimulation (∼20 s), the photocurrent through the channel seemed to remain constantly high (Berndt et al., 2009); however, whether this is maintained at higher stimulation durations (i.e., ∼60–95 s) is to be investigated.

We also observed in this study, as an effect of optogenetic stimulation in vivo, that there was an increase in CBF baseline. This could be due to the vascular response to astrocytic stimulation, which has been shown to take longer than the resting time between stimulations with the 20% stimulation paradigm (Masamoto et al., 2015; Suarez et al., 2025). Astrocytic calcium changes can trigger a complex surge of vasoactive signals, including the release of K^+^ to the perivascular space, which have direct implications on the vessel tone. This process is slow and sustained, and possibly since the blood vessels have not returned to their resting diameter before the next stimulation pulse is on, the relative CBF response for the succeeding pulses is smaller than the first. We observe that after the third stimulation pulse, the peak of the CBF response seems to be saturated. This could indicate that peak vessel dilation was attained with our stimulation paradigm but needs further investigation via simultaneous studies of calcium and LDF/blood vessel imaging to delineate the exact time course of these events.

For the acute brain slice calcium imaging experiments in this study, we found that a power density of 7 μW/mm^2^ (whole slice illumination) was sufficient to reliably elicit calcium responses in astrocytes without inducing photodamage/photobleaching. The stimulation protocol was optimized empirically based on signal reproducibility and stability. Additionally, at this low power setting in brain slices, we also found that consistently higher stimulation paradigms/duty cycles (e.g., δ_blue_ of 80 and 95%) led to saturation of astrocytic calcium responses. However, for the in vivo CBF experiments, the light was more spatially focused, illuminating a roughly circular region of ∼5 mm diameter, as confirmed and documented in our recent publication (Suarez et al., 2025). The optical power reaching the tissue was 3 mW, which corresponds to a power density of ∼0.15 mW/mm². While this power density is lower compared with other published studies (Masamoto et al., 2015; Shen et al., 2017; Tan et al., 2017), it was adopted after testing different light intensities, as shown in Suarez et al. (2025), until we identified the lowest effective power that elicited a reliable blood flow response. This approach was adopted to minimize the risk of nonspecific or phototoxic effects while ensuring sufficient activation of astrocytic pathways. Our goal was to selectively engage astrocytic calcium responses and monitor the resulting vascular dynamics LDF. The efficacy of this stimulation protocol was confirmed by consistent, reproducible astrocyte-induced blood flow changes across trials. It is crucial to note that the choice of higher power for the LDF experiments as compared with the slice experiments in this was also due to the higher depth of light penetration necessary to induce a secondary response like CBF, as compared with more surface-level astrocytic calcium imaging in acute brain slices. Also, highly synchronized astrocytic activity is required to evoke a CBF response, while the focus was only in single cell responses for calcium imaging.

It is important to note that usage of a two-photon microscope would aid in the demarcation of calcium responses from astrocytic soma versus processes and would facilitate recording for longer periods of time with reduced photobleaching. Longer recording times would provide more information for various other T-δ combinations and help understand the effect of a rest period after a stimulation cycle, even in cases of 95%. For example, a resting/dark period after 1–2 stimulations could help revive the lowering of spiking as observed in Figure 3*D*, if there was enough time for ChR2(C128S) to go to the dark adaptive state D470 (Fig. 1). Additionally, it would also be important to investigate stimulation paradigms with duty cycles below 20% while using a higher power setting on the blue light LED. At this given power, we did not observe any calcium changes in astrocytic calcium with duty cycles <20%, but this could be an avenue to explore using higher power, lower duty cycles to expand on our findings. We do not expect phototoxicity or saturation of astrocytic calcium levels with duty cycles <20% and higher power, also predicted in silico by Moshkforoush et al. (2021). Overall, this work serves to set the foundation for characterizing astrocytic calcium responses to various light stimulation paradigms, studying the implications of light stimulation to achieve regulated and controlled manipulation of astrocytes using optogenetics.

## Data Availability

All data generated or analyzed during this study are included in this published article (and its extended data files).

## Synthesis

Reviewing Editor: Harald Sontheimer, University of Virginia Decisions are customarily a result of the Reviewing Editor and the peer reviewers coming together and discussing their recommendations until a consensus is reached. When revisions are invited, a fact-based synthesis statement explaining their decision and outlining what is needed to prepare a revision will be listed below. The following reviewer(s) agreed to reveal their identity: Shan Jiang.

there are several minor concerns that need to be addressed, as listed below:

1. On page 3, "we identified paradigms 20%, 40% and 60% (of T=100s) to elicit ..." the authors need to clarify what the 20%, 40%, and 60% are here. Otherwise, it could be easily confused with the percentage of maximum power. Also, "T" needs to be clearly defined when it appears in the text for the first time. (It was only defined in the caption of Fig.1.)

2. What are the reasons for choosing the power of 500µW in the optogenetic stimulation on brain slices, and the power density of 0.15mW/mm^2 for the optogenetic stimulation in vivo? 0.15mW/mm^2 seems quite small compared to those used in published literature.

3. What is the expected effect of optogenetic stimulation with duty cycles below 20% (e.g. 10%)? Can the stimulation be more effective than the 20%? Either some additional experiments need to be added to conclude that 20% is optimal, or perhaps some discussions need to be added in terms of why 20% is a good starting point. Is it future direction to explore light stimulation effects with <20% duty cycle?

4. Fig 3D is missing its label in Fig.3. Similarly, Fig 4D is missing the label.

5. Several grammar mistakes need to be corrected in the text. For example, on page 3, "...demonstrated minimally regarding..." is confusing. On page 3, "...both rapid and slow responses responses.." is repetitive.

## Author Response

Thank you for your meticulous review of our manuscript. Please find below our responses to the questions.

1. On page 3, "we identified paradigms 20%, 40% and 60% (of T=100s) to elicit ..." the authors need to clarify what the 20%, 40%, and 60% are here. Otherwise, it could be easily confused with the percentage of maximum power. Also, "T" needs to be clearly defined when it appears in the text for the first time. (It was only defined in the caption of Fig.1.) Response: Thank you for the recommendation. We have added the definitions for T, δ and what we define as a paradigm in the introduction of the manuscript, methods and results sections as well (highlighted in yellow). The following are the sentences we have added in the introduction- "In this study, T denotes the pulse period of light stimulation and δblue (for blue light) and Δ (for Amber light) denote pulse widths within T/ duty cycle for which light is on (Fig. 1). We define a paradigm to be the value of δ within the duration of T =100 seconds for which the blue light is on (20% paradigm/ duty cycle refers to δ of 20s/ 20% of T (100s)." 2. What are the reasons for choosing the power of 500µW in the optogenetic stimulation on brain slices, and the power density of 0.15mW/mm^2 for the optogenetic stimulation in vivo? 0.15mW/mm^2 seems quite small compared to those used in published literature.

Response: Thank you for your thoughtful observation regarding the stimulation parameters. We would like to clarify the rationale and experimental considerations for the chosen values by separating the two different experimental paradigms.

Firstly, we are converting these parameters to be comparable. Masamoto et al 1 defined two power ranges for stimulation: low (0.1-0.3 mW) and high (2.8-3.3 mW) and tightly focused the illumination onto 0.2 mm2. Therefore, the power density ranged between 0.5 mW/mm2 (lowest) and 16.5 mW/mm2 (highest). The high-power induced concentric propagating waves of cerebral blood flow (CBF) responses and the authors discussed several scenarios for propagating CBF responses. Other studies by Tan et al 2 (in vivo preparations) used high power densities of 8-12 mW/mm2 and Shen et al 3 in acute brain slice preparations used 0.9-6.5 mW/mm2.

To reduce photobleaching and photo toxicity we decided to use lower power density in our experiments but ensuring that the astrocytic calcium and cerebral blood flow responses were elicited. For the in vivo experiments in this study, we have used a power density of 0.15 mW/mm², which was demonstrated in a recent publication from our lab by Suarez et al 4 to be sufficient to generate a physiologically relevant CBF responses. For the acute brain slice experiments in this study, we found that a power density of 7μW/mm2 (whole slice illumination - calculated by dividing the total power (500 μW) by the illuminated area (π × (4.75 mm)² ≈ 70.8 mm²)) was sufficient to reliably elicit calcium responses in astrocytes without inducing photodamage/photobleaching. The stimulation protocol was optimized empirically based on signal reproducibility and stability. Additionally, at this low power setting in brain slices, we also found that consistently higher stimulation paradigms/ duty cycles (e.g. δblue of 80% and 95%) led to saturation of astrocytic calcium responses. As pointed out by the reviewers, various power densities and their impact on astrocytic calcium needs to be evaluated in future work, since in this current study, we focused on the temporal profile of astrocytic stimulation and associated calcium and CBF responses.

We have modified the manuscript to clarify this in the methods section and added a paragraph in the discussion section highlighted in yellow.

3. What is the expected effect of optogenetic stimulation with duty cycles below 20% (e.g. 10%)? Can the stimulation be more effective than the 20%? Either some additional experiments need to be added to conclude that 20% is optimal, or perhaps some discussions need to be added in terms of why 20% is a good starting point. Is it future direction to explore light stimulation effects with <20% duty cycle? Response: Thank you for giving us a chance to expand on this. At the power density of 7μW/mm2, our preliminary experiments with duty cycles <20% of blue light stimulation in acute brain slice preparations did not show any changes in astrocytic calcium levels (measured by changes in Rhod-2 AM fluorescence). It is definitely a future direction to investigate at a higher power level, lower duty cycles (<20%) to check for changes in astrocytic calcium. We do not expect phototoxicity or saturation of astrocytic calcium levels with duty cycles <20%, also predicted in silico by a previous study by our group 5. We have added a short write up in the discussion/ future directions to explore about this (in yellow highlights). Our goal for this study was to use the lowest power setting possible in order to not cause drastic increases in temperature, while leading to astrocytic calcium responses as an effect of optogenetic stimulation.

4. Fig 3D is missing its label in Fig.3. Similarly, Fig 4D is missing the label.

Response: Thank you for noticing this. We have added the panel labels, which are reflected now in the word and pdf versions.

5. Several grammar mistakes need to be corrected in the text. For example, on page 3, "...demonstrated minimally regarding..." is confusing. On page 3, "...both rapid and slow responses responses.." is repetitive.

Response: Thank you for letting us know about the grammatical errors. We have corrected them and highlighted the changes in yellow.

REFERENCES 1 Masamoto, K. et al. Unveiling astrocytic control of cerebral blood flow with optogenetics. Scientific reports 5, 11455 (2015).

2 Tan, Z. et al. Glia-derived ATP inversely regulates excitability of pyramidal and CCK-positive neurons. Nature communications 8, 13772 (2017).

3 Shen, W., Nikolic, L., Meunier, C., Pfrieger, F. &Audinat, E. An autocrine purinergic signaling controls astrocyte-induced neuronal excitation. Scientific reports 7, 11280 (2017).

4 Suarez, A., Fernandez, L. &Riera, J. Characterizing astrocyte-mediated neurovascular coupling by combining optogenetics and biophysical modeling. Journal of Cerebral Blood Flow &Metabolism, 0271678X241311010 (2025).

5 Moshkforoush, A., Balachandar, L., Moncion, C., Montejo, K. A. &Riera, J. Unraveling ChR2-driven stochastic Ca2+ dynamics in astrocytes: A call for new interventional paradigms. PLoS Computational Biology 17, e1008648 (2021).
