# Supplementary figures and images for: Characterization of Optimal Optogenetic Stimulation Paradigms to Evoke Calcium Events in Cortical Astrocytes
Source: eNeuro. 2025 Sep 12;12(9):ENEURO.0220-25.2025. doi: 10.1523/ENEURO.0220-25.2025 (PMC12440239; doi:10.1523/ENEURO.0220-25.2025)

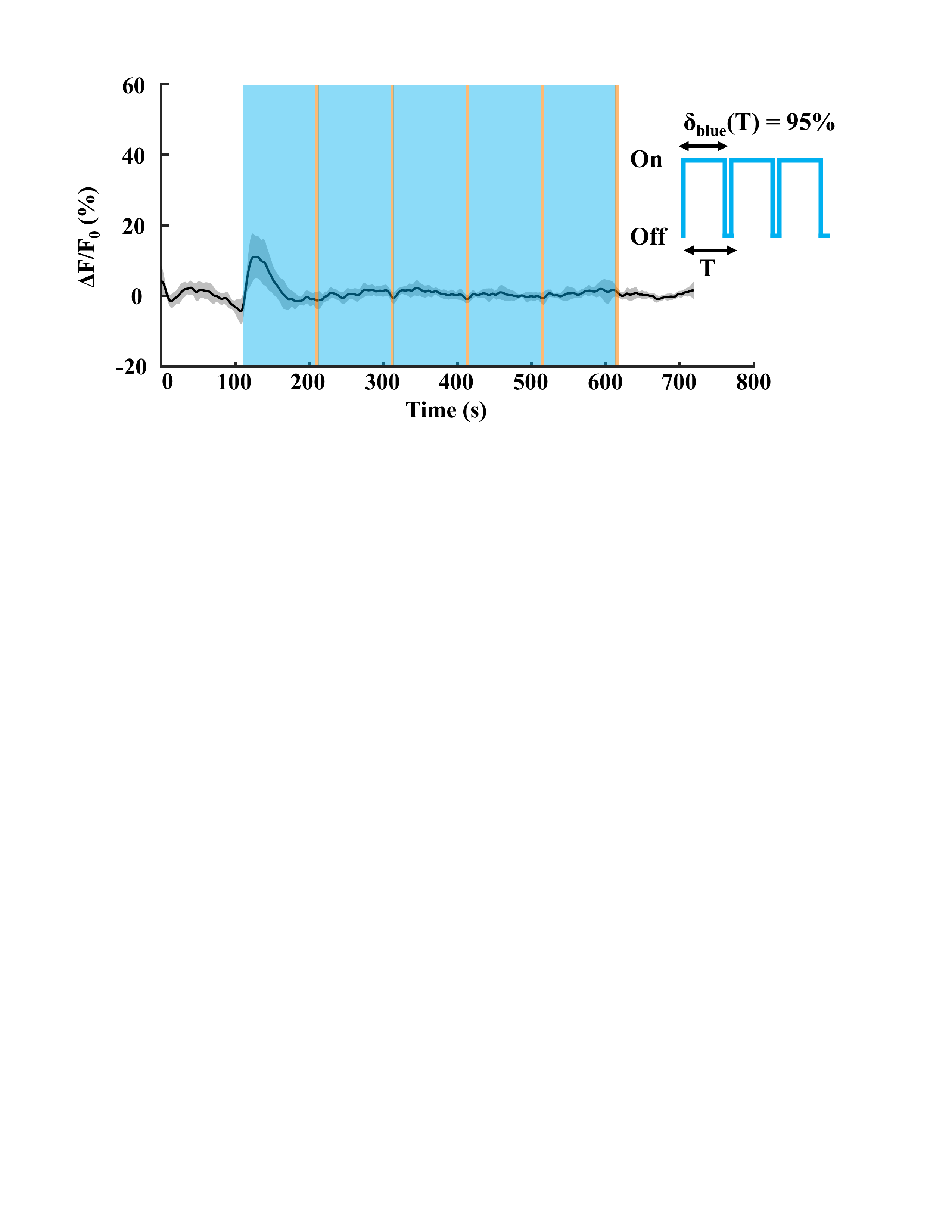

Supplement: Figure 3-1 — Astrocytic calcium responses to near continuous light stimulation. Download Figure 3-1, TIF file. [file eneuro-12-ENEURO.0220-25.2025-s001.tif]
